# Expression and function of Abcg4 in the mouse blood-brain barrier: role in restricting the brain entry of amyloid-β peptide

**DOI:** 10.1038/s41598-017-13750-0

**Published:** 2017-10-17

**Authors:** Agnès Dodacki, Matthew Wortman, Bruno Saubaméa, Stéphanie Chasseigneaux, Sophie Nicolic, Nathalie Prince, Murielle Lochus, Anne-Laure Raveu, Xavier Declèves, Jean-Michel Scherrmann, Shailendra B. Patel, Fanchon Bourasset

**Affiliations:** 1 Variabilité de Réponse aux Psychotropes, INSERM, U1144 Paris, France; 2 Faculté de Pharmacie de Paris, Université Paris Descartes, UMR-S 1144 Paris, France; 3 Université Paris Diderot, UMR-S 1144 Paris, France; 4 Division of Endocrinology, Diabetes and Metabolism, University of Cincinnati, Cincinnati, OH USA

## Abstract

ABCG4 is an ATP-binding cassette transmembrane protein which has been shown, *in vitro*, to participate in the cellular efflux of desmosterol and amyloid-β peptide (Aβ). ABCG4 is highly expressed in the brain, but its localization and function at the blood-brain barrier (BBB) level remain unknown. We demonstrate by qRT-PCR and confocal imaging that mouse Abcg4 is expressed in the brain capillary endothelial cells. Modelling studies of the Abcg4 dimer suggested that desmosterol showed thermodynamically favorable binding at the putative sterol-binding site, and this was greater than for cholesterol. Additionally, unbiased docking also showed Aβ binding at this site. Using a novel *Abcg4*-deficient mouse model, we show that Abcg4 was able to export Aβ and desmosterol at the BBB level and these processes could be inhibited by probucol and L-thyroxine. Our assay also showed that desmosterol antagonized the export of Aβ, presumably as both bind at the sterol-binding site on Abcg4. We show for the first time that Abcg4 may function *in vivo* to export Aβ at the BBB, in a process that can be antagonized by its putative natural ligand, desmosterol (and possibly cholesterol).

## Introduction

The brain capillary endothelial cells (BCECs) represent the main interface separating the blood from the brain at the blood–brain barrier (BBB)^1,2^. The permeability restriction of the BBB (comprising of the basement membrane, pericyte and astrocyte foot-processes) is mainly due to the presence of tight junctions (TJ) connecting the BCECs to each other^3,4^. Several influx and efflux transporters, such as solute carriers (SLC) and ATP-binding cassette (ABC) proteins are expressed in these BCECs^1^. ABC transporters belong to a superfamily of transmembrane proteins, divided into 7 sub-families (A to G) according to their structural homology^5–7^. The most studied ABC transporters at the BBB are ABCB1 (P-glycoprotein, P-gp) and ABCG2 (breast cancer resistant protein, BCRP), which efflux their substrates, mainly exogenous compounds, out of the brain into the bloodstream^1,2,8^. Besides ABCB1 and ABCG2, other ABC transporters belonging to ABCA and ABCG subfamilies are also expressed at the BBB^1–3^. Since ABCA and ABCG family members are involved in the transport of lipids, their potential expression at the BBB level would lend support to the concept that they may help regulate brain-lipid homeostasis. However, little is known about their exact localization and roles. ABCA1 mRNA and protein have been detected in cultured human and rat BCECs, as well as in isolated mouse microvessels^9,10^. We showed previously that Abca1, a full-transporter, acts as an efflux transporter at the BBB level and that probucol, a known inhibitor of ABCA1^11^, inhibited cholesterol efflux at the luminal side of murine BCECs^10^. ABCG proteins are half-transporters that dimerize with other half-transporters to become functional^12–14^. Hence, ABCG2 and ABCG4 forms homodimers, though ABCG4 can, *in vitro*, form homo- or hetero-dimers with ABCG1, but not with ABCG2^12,15,16^. Whether ABCG4 exists as a homodimer or as heterodimer *in vivo* has not been established. ABCG4 and ABCG1 are highly expressed in the brain, in particular in neurons, microglia and astrocytes^17^. We have previously shown that Abcg4 is expressed in micro-vessels isolated from mouse brains^18^. ABCG4 may be involved in the cellular efflux of sterols, in particular cholesterol and desmosterol (a cholesterol precursor), to high-density lipoprotein (HDL)^17,19,20^. Therefore, ABCG4 could play a role in Alzheimer’s disease (AD)^21,22^, where normal cholesterol metabolism is also disrupted. In AD, excessive accumulation and aggregation of amyloid-β peptide (Aβ) is observed in the brain^23^. Aβ originates from the amyloid precursor protein (APP) which is cleaved by both the β- and γ-secretases to produce Aβ in the brain^24^. Supporting the hypothesis that ABCG4 is involved in AD pathology, Uehara *et al*. showed ABCG4 was over-expressed in microglia surrounding senile plaques^25^. More recently, ABCG1 and ABCG4 were shown to modify the plasma membrane distribution of the γ-secretase, leading to a decrease in its activity, with a reduction of Aβ production^26^. However, for the vast majority of late-onset AD, where production rates of Aβ seem unaffected, the major defect is the impaired clearance rate of Aβ, especially at the BBB level^18,27–31^. ABCB1 and ABCG2, as well as the low density lipoprotein receptor-related protein 1 (LRP1) have also been shown to be involved in the BBB efflux of Aβ, though other unidentified transporters are also likely to be involved in Aβ export^8,18,32,33^. In AD, Aβ mainly exists as 40 or 42 amino-acids forms, and both are thought to be transported by the same set of transporters^34–36^. In addition, Aβ_1–40_ is more stable in solution than Aβ_1–42_^37,38^, thus, we have studied the transport mechanisms of Aβ_1–40_^18^. Our prior studies have shown that Aβ_1–40_ was actively transported by the murine Abcg4 from intra- to extra-cellular compartment of HEK-*Abcg4* transfected cells and that probucol specifically inhibited this Abcg4-mediated cell efflux^18^. Whether Abcg4 carries out this function *in vivo* remains to be established.

The aim of this study is to characterize the function of Abcg4 at the mouse BBB to test the hypothesis that it can export desmosterol and Aβ, and characterize the interplay between these two ligands using an *in vivo* model.

## Results

### Immunodetection of Abcg4 in wild-type (WT) mice

**Figure 1:**
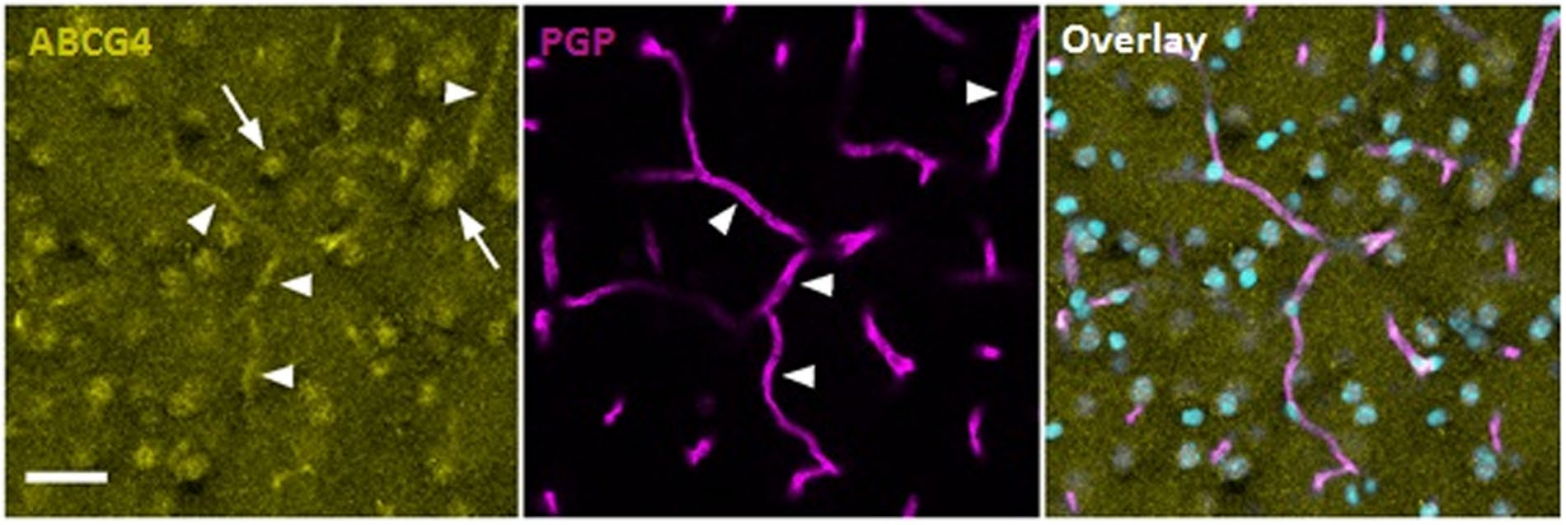
Immunodetection of Abcg4 in wild-type (WT) mice. Brain capillaries are identified by endothelial cell-specific P-gp labelling (magenta, middle panel) and nuclei are counterstained by Topro3 (cyan). Abcg4 staining (yellow, left hand panel) is localized to parenchymal cells (arrows) and endothelial cells of the capillaries (full arrowheads). The overlay image of all three (right hand panel) indicates co-localization of Abcg4 with P-gp in the brain capillaries. Scale bar: 30 µm.

Abcg4 and Abcb1 (also known as P-gp or Mdr1) co-immunostaining of WT mouse brain cryosections were performed to assess the presence of the transporters at the BBB (Fig. 1). Abcg4 labelling was detected in parenchymal cells and in P-gp-positive brain cortex capillaries. No detectable signal could be observed when primary antibody was omitted (not shown). The parenchymal staining was expected, since it has been shown previously that Abcg4 was expressed in neurons and astrocytes^17,39^. Our data demonstrate that Abcg4 staining was also present in brain capillaries, supporting its role as a BBB transporter.

### Expression patterns of other ABC transporters in WT and Abcg4-knockout (KO) mice BBB

We compared, by qRT-PCR, the relative mRNAs of the ABC transporters involved in sterol and/or Aβ efflux through the mouse BBB (*Abca1*, *Abcb1*, *Abcg1*, *Abcg2* and *Abcg4)*.

**Figure 2:**
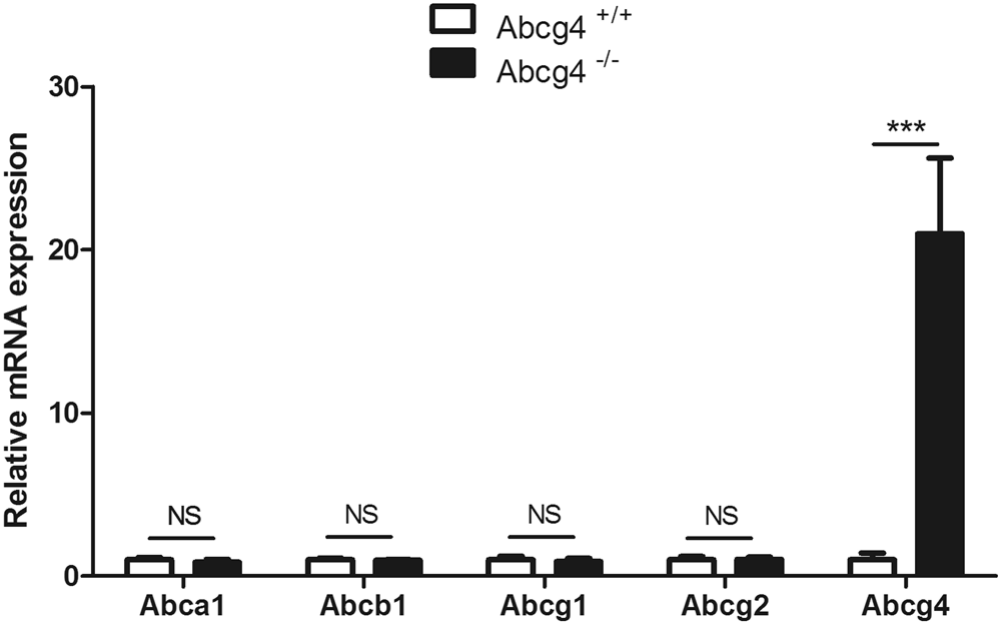
Quantification of transcripts of several ABC transporters in wild-type (WT) and *Abcg4*-KO mice BBB. The mRNA levels of each gene were determined by qRT-PCR and normalized to those of β-actin as described in Methods. Data are means ± S.D. (n = 3 experiments). *p < 0.05 (Student’s unpaired *t* test).

Expression of *Abca1*, *Abcb1*, *Abcg1 and Abcg2* were not significantly different in micro-vessels harvested from *Abcg4*-KO mice compared to WT mice (Fig. 2). Although the expression of *Abcg4* was increased by more than 20-fold, the primers used are located at the 5′ end of the gene: primers at the 3′ end, after the targeted disruption, did not amplify any product (although aberrant splicing was detected in the *Abcg4*-KO mice, see Methods). The basis for the compensatory increased transcription in the *Abcg4*-KO mouse was not studied herein.

### Abcg4 function at the mouse BBB

The function of Abcg4 at the mouse BBB was evaluated by using the *in situ* brain perfusion (ISBP) technique. This method was developed to study drug transport processes occurring at the luminal side of the BCECs^40,41^ and can be used to measure transport parameters, including the brain volume of distribution and the brain uptake clearance (Cl_up_) of a target molecule over very short periods (30–120 s). The resulting Cl_up_ integrates the effect of enhancing or limiting factors introduced by the transporters located at the luminal side of BCECs. This method allows for measurement of the initial brain uptake of molecules, which results from both the influx and the efflux mechanisms involved in their transport. One of the advantages of this technique is the ability to administer inhibitors, or to use transporter deficient mice, in order to modulate function at the luminal side of the BBB. Hence, if the Cl_up_ increases when a transporter inhibitor is added or when a transporter is missing, we can conclude that the transporter concerned is an efflux carrier involved in the luminal BBB transport of the target molecule. On the other hand, if the Cl_up_ decreases when a transporter inhibitor is added or when a transporter is missing, we can conclude that the transporter concerned is an influx carrier involved in the luminal BBB transport of the target molecule.

**Figure 3:**
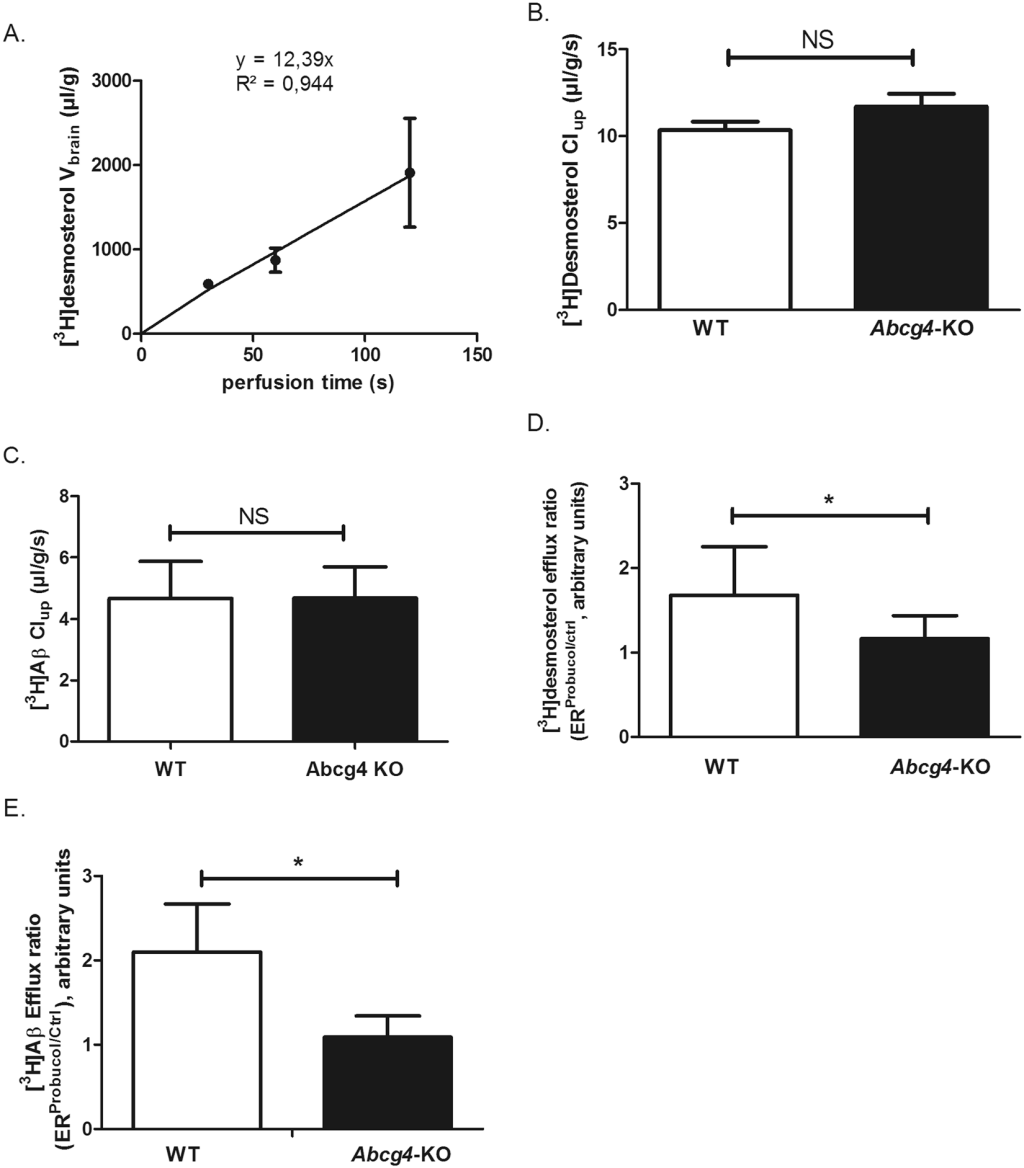
Abcg4 function at the mouse BBB. Panel A shows the time course of [^3^H]desmosterol uptake by the right hemisphere of C57BL/6 mice, expressed as apparent volume of distribution (V_brain_, µl/g brain), determined by *in situ* brain perfusion technique, r^2^ = 0.944 for regression analysis of individual data. Data are means ± S.D. of 3 animals per data point. Panels B shows the brain uptake clearance (Cl_up_, µL/g/s) of [^3^H]desmosterol and Panel C for [^3^H]Aβ_1–40_ (C) in WT and *Abcg4*-KO mice, measured by *in situ* brain perfusion technique. Panel D shows the efflux ratio (ER) measured in WT and *Abcg4*-KO mice: ratio for [^3^H]desmosterol and panel E that for [^3^H]Aβ_1–40_, Cl_up_ obtained in the presence or absence of probucol (10 µM) (ER^Probucol/Ctrl^). Panels B-E: Data are means ± S.D. of 8–9 mice. *p < 0.05, NS non-significant (two-way ANOVA analysis followed by a Bonferroni post-test).

We measured first the time-course of the brain volume of distribution (V_brain_, µL/g) of [^3^H]desmosterol in WT mice (Fig. 3A) and observed a linear increase of [^3^H]desmosterol V_brain_ as a function of the perfusion time, suggesting that [^3^H]desmosterol transport rate remained unchanged over time. We chose a perfusion time of 60 s for subsequent single time-points experiments, based upon this and our prior work using [^3^H]Aβ_1–40_^18^.

We evaluated the function of Abcg4 at the BBB level by measuring the brain uptake clearance (Cl_up_) of [^3^H]desmosterol and [^3^H]Aβ_1–40_, in WT and *Abcg4*-KO mice, in the presence or absence of probucol. In the absence of probucol, the [^3^H]desmosterol and [^3^H]Aβ_1–40_ Cl_up_ obtained in *Abcg4*-KO mice were not significantly different from those in WT mice (Fig. 3B and C), suggesting either that Abcg4 was not involved in their efflux at the luminal side of BCECs or that the lack of Abcg4 has been offset by other efflux proteins at the mouse BBB, like Abcg1, for desmosterol^17^ or Abcb1/Abcg2 for Aβ^18^. However, in the presence of probucol, [^3^H]desmosterol and [^3^H]Aβ_1–40_ Cl_up_ were significantly increased in WT mice, 1.8- and 2.1-fold respectively, as illustrated by the [^3^H]Aβ_1–40_ efflux ratio calculated in the presence or absence of probucol (ER^probucol/ctrl^, Fig. 3D and E), but this effect was lost in *Abcg4-*KO mice. Probucol has been shown to inhibit both ABCA1^11^ and ABCG4^18^ but not ABCG1^42^, indicating that probucol had inhibited an Abcg4-mediated efflux of [^3^H]desmosterol and [^3^H]Aβ_1–40_ at the luminal BCECs level in WT mice. These results support the premise that Abcg4 is an efficient exporter, at the BBB level, of desmosterol and Aβ.

### L-thyroxine (T_4_) inhibition of Abcg4-mediated efflux of [^3^H]Aβ_1-40_ and [^3^H]desmosterol at the BBB level

**Figure 4:**
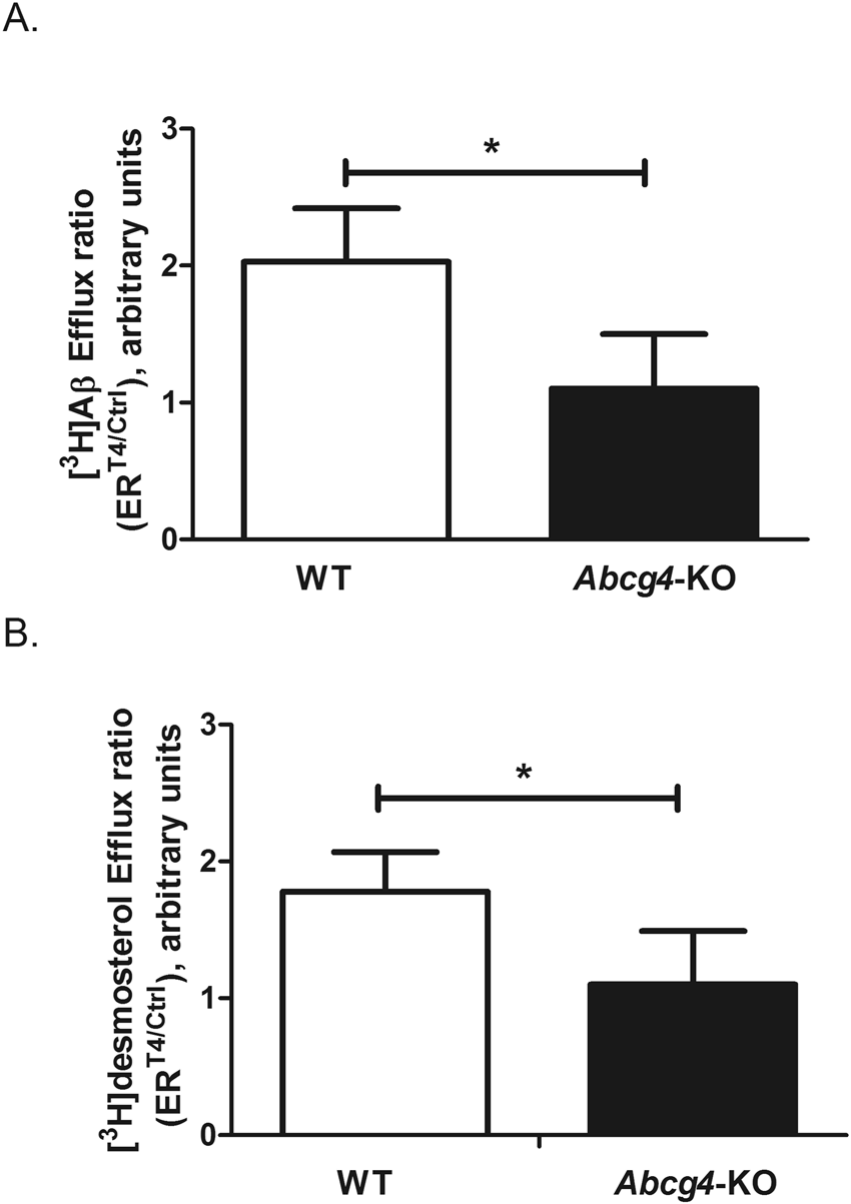
L-thyroxine (T_4_) inhibition of Abcg4-mediated efflux of [^3^H]Aβ_1–40_ and [^3^H]desmosterol at the mouse BBB level. Efflux ratio of [^3^H]desmosterol (Panel A) or [^3^H]Aβ_1–40_ (Panel B) obtained by dividing the brain uptake clearance (Cl_up_, µL/g/s) measured in the presence of T_4_ (10 µM) over the Cl_up_ measured in the absence (Ctrl) of T_4_ (ER^T4/ctrl^) in WT and *Abcg4-*KO mice. Data are means ± S.D. of 5–8 mice. *p < 0.05 (two-way ANOVA analysis followed by a Bonferroni post-test).

We had demonstrated previously that T_4_ increased the brain uptake of Aβ by inhibiting one or more unidentified efflux transporters located at the luminal side of mouse BCECs^43^. This process could potentially involve Abcg4. We measured the Cl_up_ of [^3^H]Aβ_1–40_ and [^3^H]desmosterol in the presence or absence of T_4_ in WT and *Abcg4*-KO mice. Adding T_4_ to the perfusion buffer significantly increased the Cl_up_ of [^3^H]desmosterol and [^3^H]Aβ_1–40_ in WT mice (1.8-fold and 2-fold, respectively, Fig. 4), but this effect was lost in *Abcg4*-KO mice. Taken together, these data demonstrate that T_4_ inhibited the Abcg4-mediated efflux of [^3^H]Aβ_1-40_ and [^3^H]desmosterol from BCECs to blood.

### **In vivo** interaction between [^3^H]Aβ_1–40_ and desmosterol at the mouse BBB level

**Figure 5:**
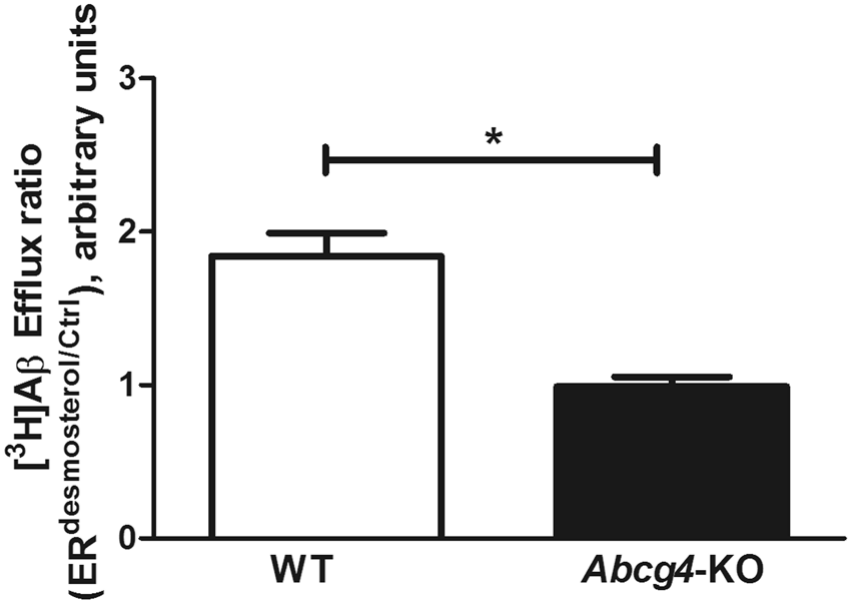
*In vivo* interaction between [^3^H]Aβ_1–40_ and desmosterol at the mouse BBB level. Efflux ratio of [^3^H]Aβ_1–40_ obtained by dividing the brain uptake clearance (Cl_up_, µL/g/s) measured in the presence of desmosterol (20 µM) over the Cl_up_ measured in the absence (Ctrl) of desmosterol (ER^desmosterol/ctrl^) in WT and *Abcg4-KO* mice. Data are means ± SD of 5–7 mice. *p < 0.05 (two-way ANOVA analysis followed by a Bonferroni post-test).

We tested whether desmosterol and [^3^H]Aβ_1–40_ could compete for Abcg4-mediated efflux, or whether these were independent processes. We measured the Cl_up_ of [^3^H]Aβ_1–40_ in the presence or absence of desmosterol in WT and *Abcg4*-KO mice. Adding desmosterol to the perfusion buffer increased significantly the Cl_up_ of [^3^H]Aβ_1-40_ in WT mice (1.8-fold) while no significant effect was observed in *Abcg4*-KO mice, as illustrated by the [^3^H]Aβ_1-40_ efflux ratio calculated in the presence or absence of desmosterol (ER^desmosterol/ctrl^, Fig. 5). These data demonstrate that desmosterol inhibits the Abcg4-mediated efflux of [^3^H]Aβ_1-40_ from BCECs to blood.

### Abcg4 homology modeling

**Figure 6:**
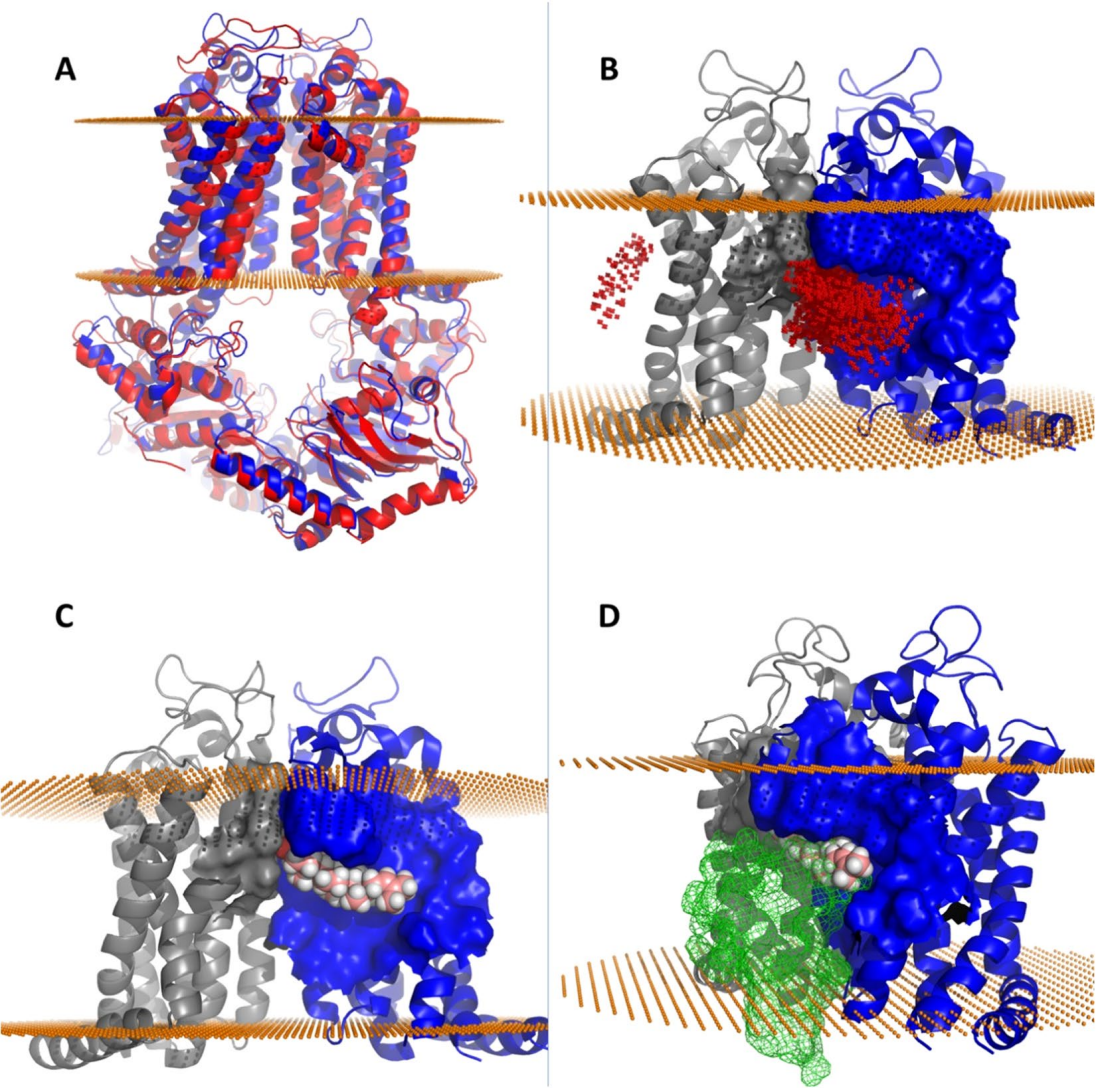
Computed structural and binding features of the Abcg4 dimer model. Mouse Abcg4 sequence was submitted to the ModBase modeling server which constructed an Abcg4 model (blue) after selecting the x-ray crystal structure of ABCG5/ABCG8 (red cartoon) as the template in an unbiased search (Panel A). Panel B shows docking of desmosterol across the entire transmembrane surface identified two putative desmosterol binding sites (red dots represent the placement of desmosterol after each docking run). The overwhelming majority of final binding modes for desmosterol on Abcg4 were located in the homologous sterol binding cleft found in ABCG5/ABCG8 suggesting conserved sterol binding domain is found in both dimers. Best predicted binding mode of desmosterol (pink and white spheres) in the cleft formed by Abcg4 dimers (gray and blue, Panel C). Gray monomer rotated 90° away from the viewer along the axis of symmetry reveals competitive binding between desmosterol and Aβ (green mesh, panel D).

Although we have biochemical evidence for the role of Abcg4 in desmosterol and Aβ efflux, we asked if there is physical support for this function. A homology model of the Abcg4 dimer, consisting of residues 82–641, was created using the published x-ray structure of a related ABCG protein complex ABCG5/ABCG8^44^ that is also known to transport sterols (Fig. 6A). The initial model was optimized by adding missing side-chain atoms, relaxing steric clashes, and sampling rotomers to identify a favorable configuration. Overall model quality, as assessed by MolProbity^45,46^, increased from the 23^rd^ to the 92^nd^ percentile (N = 27675, 0 Å–99 Å). While a small segment of residues from the protein sequence termini could not be modeled, a significant portion had sufficient homology to develop a model that includes the nucleotide-binding domain, the transmembrane domain including the sterol binding site^44^, and the extracellular region. Based on the structure and purported mechanism of the related ABCG5/ABCG8 protein complex, sterols bind to a cavity formed at the dimer interface that allows sterols to move from the lipid bilayer into the dimer core followed by translocation to the membrane surface^44^. Analogous structural features present in our model suggest that the Abcg4 dimer may translocate sterols by an analogous mechanism (Fig. 6B, C and D).

### Sterol docking

We tested the feasibility of desmosterol translocation in the Abcg4 model by performing 15,000 simulations of desmosterol binding to Abcg4 using SwissDock^47^ in accurate mode with no side-chain flexibility (Fig. 6B). While the structure of ABCG5/ABCG8 places the sterol-binding site in a cleft formed at the dimer interface, we chose to allow SwissDock to dock desmosterol over the entire dimer surface to avoid bias. Importantly, of the top 250 predicted binding modes, all but one were found in the predicted sterol binding cleft and all had favorable binding energetics with ΔG between −6.79 and −8.12 kcal/mol. In addition, simulations using cholesterol produced nearly identical results, though with slightly less favorable binding energetics (ΔG between −6.14 and −7.79 kcal/mol) suggesting a higher affinity for desmosterol.

### Aß docking

**Figure 7:**
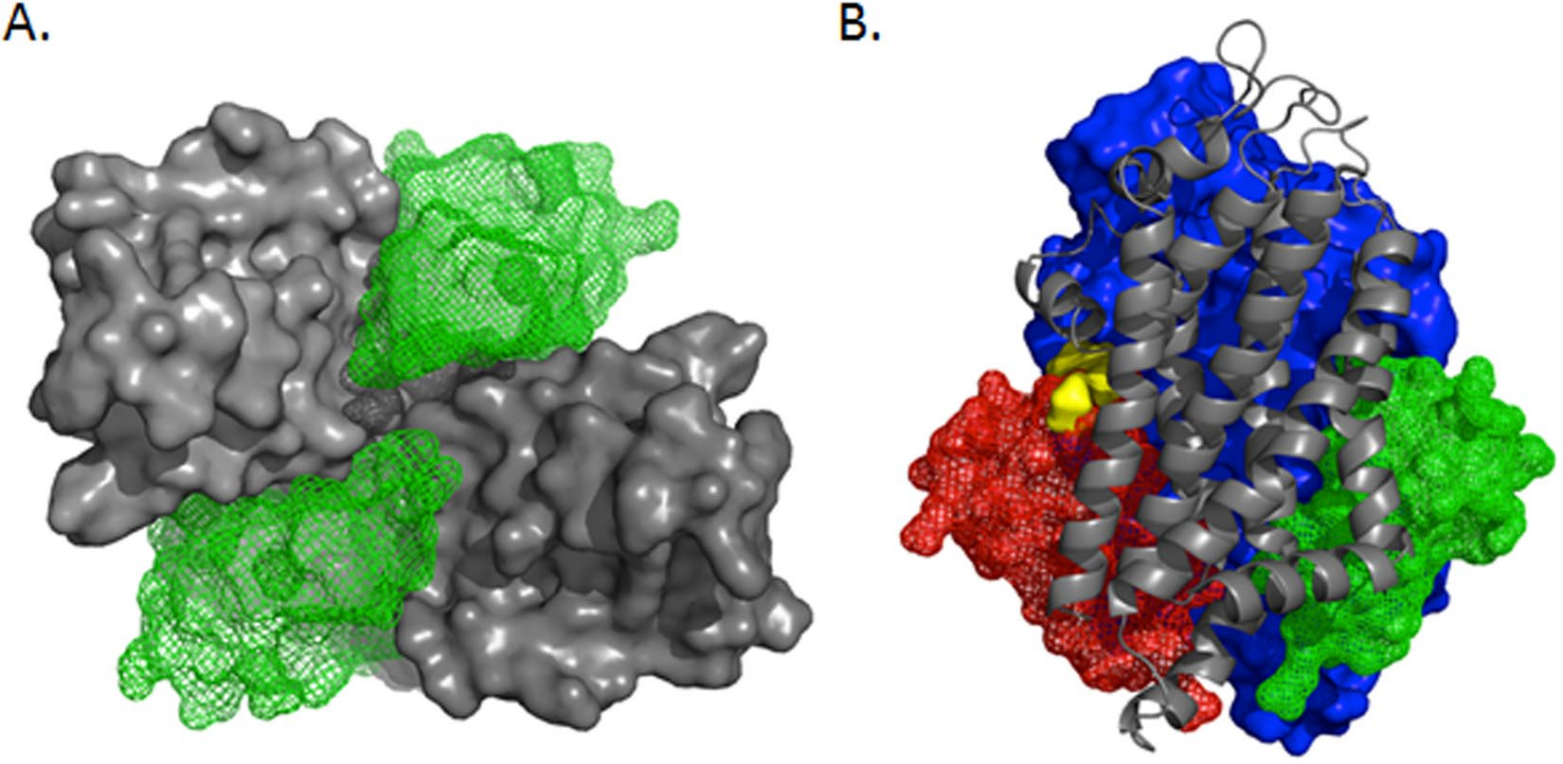
Dual Aβ binding sites in Abcg4. The Abcg4 dimer is comprised of two monomers (gray surface) that form two nearly identical binding sites for Aβ (green mesh, panel A). This view of the membrane-spanning domain from the intracellular aspect shows two Aβ molecules (green mesh) occupying the binding clefts. The membrane-spanning domain viewed from the side, panel B, shows the competitive binding mode of desmosterol (yellow surface) with Aβ (red mesh) while another Aβ molecule is present in the opposite binding cleft (green mesh). This configuration explains the observed preference for Aβ (two sites) and inhibitory effect of sterols on Aβ efflux.

Our data support a role for Abcg4 in both desmosterol and Aβ transport, despite large differences in the substrate molar mass (384.6 and 4514.1 g/mol respectively), (Fig. 7). While Aβ is not small molecule ligand, and therefore cannot be docked into a binding-site using traditional docking software, algorithms are available that predict protein:protein interactions. GRAMM-X^48,49^ is a web-based service that accepts two proteins as input and, like SwissDock for small molecules, performs a large number of unbiased simulations in order to determine a thermodynamic ‘best fit’ for a given protein pair. As with sterol docking, we did not define a binding site therefore GRAMM-X sampled the entire surfaces of both proteins. GRAMM-X also identified the dimer interface cleft as the binding site for Aβ which suggests that Aβ and desmosterol compete for binding (Fig. 7B).

Abcg4 monomers come together to form a homodimer that includes nearly identical binding clefts which are opposite one another on the dimer surface. The homology model is an approximation of the dimer structure frozen in time which fails to capture the dynamic nature of protein motions. *In vivo* it is likely that nucleotide binding changes the overall configuration of the dimer to allow for sterol binding on one side of the complex followed by translocation of sterol to the dimer core before being exported from the membrane. One consequence of this movement could be that only one sterol-binding site exists at a given time. Thus, sterol binding would alternate from one binding site to the other as sterols are translocated. This mechanism could explain why the homology model has a single sterol-binding site despite the presence of two nearly identical binding clefts. Importantly however, this does not appear to be the case for Aβ which is predicted to bind in both clefts equally (Fig. 7A). Abcg4 seems to have two Aβ binding sites which agrees with our previous data that percent efflux of Aβ was greater than cholesterol^18^ and with our current data that desmosterol competes with Aβ efflux. Furthermore, we previously measured, in HEK-*Abcg4* transfected cells, the velocity of Aβ transport by Abcg4 as a function of [^3^H]Aβ_1-40_ concentrations. The relationship was best-fitted by a Hill equation (sigmoid) with a Hill coefficient at around 2 (n = 1.80 ± 0.08), supporting the hypothesis of 2 binding sites for the Abcg4-mediated transport of Aβ. The Km was found equal to 349.9 ± 16.8 pM and the Vm to 22.4 ± 0.2 pmol/L/min. Taken as a whole, our biological and structural data suggest that Abcg4 contains two Aβ binding sites and one alternating sterol site which explains our observation that Abcg4 transports both Aβ and sterols with a preference for Aβ. While this is a plausible mechanism, more research, including direct structural observation of the Abcg4 dimer, is needed.

## Discussion

ABCG4 is expressed in neurons and astrocytes present in the hippocampus, cerebellum and olfactory bulb regions, and has been implicated in sterol trafficking in the central nervous system^17,26,39^. ABCG4 has been also detected in ependymal cells from brains taken from non-demented patients and in microglial cells from brains from subjects with Alzheimer’s disease^25,50^. The expression and function of ABCG4 in human BCECs has not been reported. We had previously detected Abcg4 in micro-vessels isolated from WT and 3xTg-AD mouse brains^18,51^, but its exact cell localization was unknown. Recently, Hegyi and Homolya reported a predominantly plasma membrane localization of ABCG4 in several cell types^15^. Furthermore, Kober *et al*. showed that ABCG1 was expressed at both luminal and abluminal sides of the porcine BCECs^52^, though they did not detect ABCG4 in these porcine BCECs, which may represent a species difference. Our current pattern of Abcg4 immunostaining and the observed co-localization with Abcb1 further suggest that Abcg4 is expressed in endothelial cells of mouse brain capillaries. Using the ISBP technique, which allows for the exploration of BBB transport mechanisms occurring at the luminal side of the BCECs^53^, we now show that Abcg4 is an efflux pump at the mouse BBB level and can export both desmosterol as well as Aβ.

**Figure 8:**
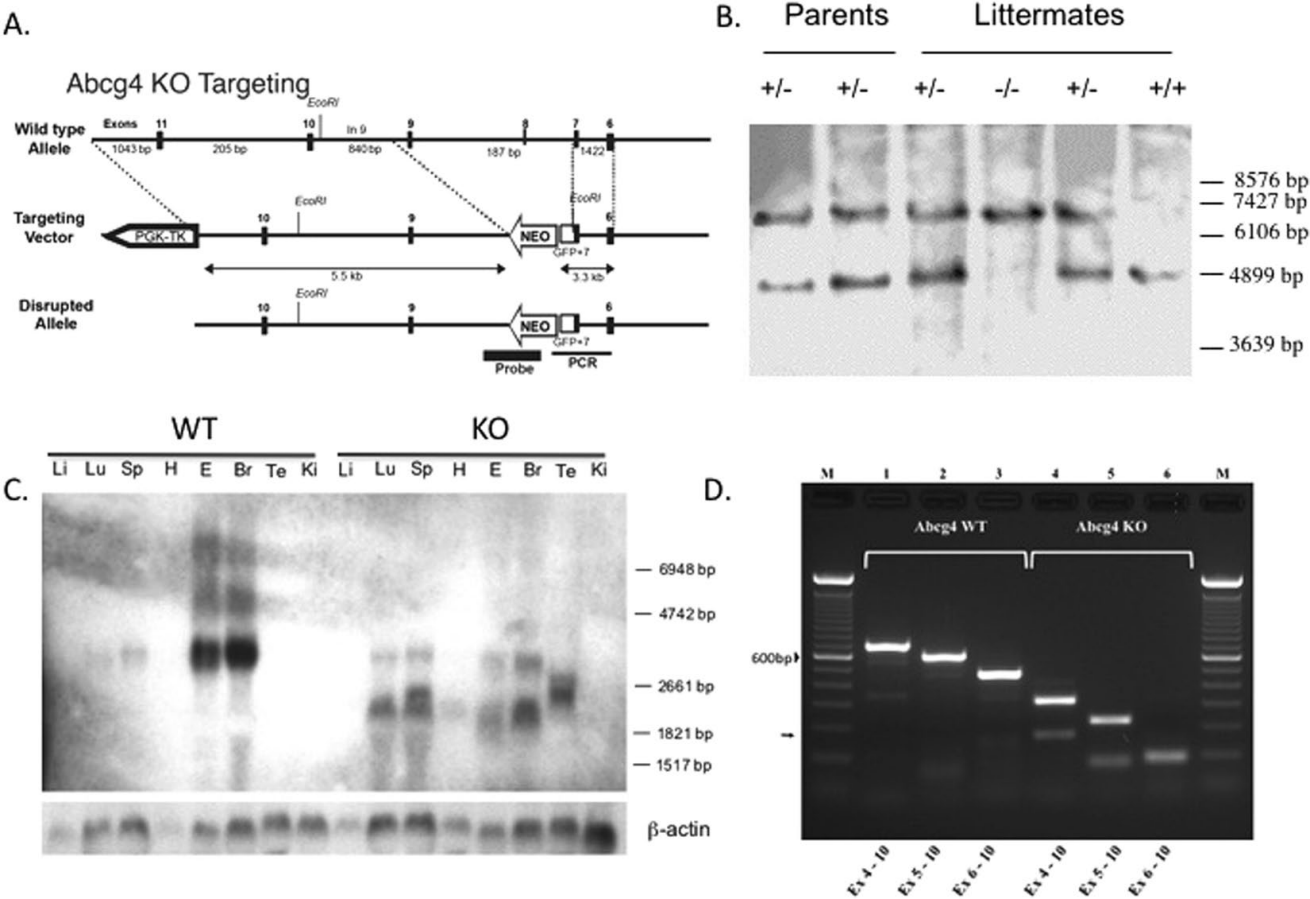
*Abcg4*-knockout mice line establishment. Panel A depicts a cartoon of the *Abcg4* knockout targeting vector used for homologous recombination. The gene structure is not drawn to scale. Targeting was engineered to result in expression of a fusion protein encoding partial *Abcg4* fused to eGFP prior to the transmembrane domains. The neomycin gene cassette is indicated by Neo. The homologous targeting removes a significant portion of coding sequences in exon 7 and all of exon 8. The Bgl I sites are as shown. Panel B shows the validation of the homologous recombination by Southern blotting. Mouse DNA was digested using Bgl I and detected using a genomic fragment spanning exons 3–5 shown in panel A. The targeted fragment (6.4 kb) is larger than the WT fragment (4.5 kb). The molecular weight marker positions were estimated from the ethidium-stained gel. Panel C shows Northern blot from WT and knockout (KO) tissues with a cDNA probe (Li: liver, Lu: lung, Sp: spleen, H: heart, E: eye, Br: brain, Te: testes, Ki: kidney). In RNA from *Abcg4*-knockout mice (‘KO’), multiple shorter transcripts were detected, compared to WT. Additionally, expression was more wide-spread, with signals detected in lung, spleen and testes, compared to WT mice. RT-PCR analyses of RNA from brains of WT (panel D, tracks 1–3) or KO (panel D, tracks 4–6) using primers in exon 4–10 (tracks 1 and 4), exon 5–10 (tracks 2 and 5) and exon 6–10 (tracks 3 and 6) led to the bona fide sized products in wild-type RNA, but these were smaller for samples from KO brain. All of the largest products, as well as one of the smaller products (arrow) were sequenced. For wild-type RNA, there was contiguous sequential exon splicing, but for *Abcg4-*KO, exon 6 was spliced to exon 10 in the largest PCR fragments and in the smaller fragment, exon 5 was spliced to exon 10 (see text for discussion).

Probucol, a known Abcg4 inhibitor^18^, was unable to modulate the brain uptake clearance (Cl_up_) of an ABCG4 substrate, [^3^H]desmosterol, in *Abcg4*-KO mice, whereas this effect was present in WT mice. Using primers designed in the non-mutated 5’ region of *Abcg4*, we showed, by qRT-PCR, that the mRNA for *Abcg4* was 20-fold increased in *Abcg4*-KO mice, as compared to WT mice. Interestingly, compensatory up-regulation has been previously observed in other KO mouse models such as *Abcg1* ^17,54^. It has been shown that *Abcg4* can be induced by the liver-X receptor (LXR) activation^17,55,56^. However, our results show that the transcription of two other LXR targets, *i*.*e*. *Abca1* and *Abcg1* ^17^, was not up-regulated in *Abcg4*-KO mice, suggesting that increased promoter activation may not involve the LXR pathway (although non-promoter effects may also be possible, such as alteration of mRNA stability). Additionally, our results show that the mRNA levels of two other ABC transporters (*Abcb1* and *Abcg2*), were also unchanged in *Abcg4*-KO mice as compared to WT mice, meaning that the absence of *Abcg4* did not perturb other ABC transporters expression. Note also (Fig. 8, panel C) that the *Abcg4*-KO led to more wide-spread expression of the truncated messages by Northern, suggesting a potential feed-back regulation of its expression. We have not investigated the molecular basis of this in our *Abcg4*-KO mice.

We had shown previously that a probucol- and T_4_-sensitive transporter, different from Abca1, Abcb1 and Abcg2, restricted the brain entry of [^3^H]Aβ_1-40_ through the mouse BBB^18,43^. In HEK-*Abcg4* transfected cells, Abcg4 was efficient at effluxing Aβ^18^ and this efflux was specifically inhibited by probucol (2-fold)^18^ and T_4_ (5.8-fold, data not shown). In the current study, we demonstrate that Abcg4 is the probucol- and T_4_-sensitive transporter involved in the restriction of Aβ brain entry at the luminal side of mouse BCECs. Our *in silico* modeling results showed that the Abcg4 dimer contains a sterol binding site that has a greater affinity for desmosterol than cholesterol and supports our current *in vivo* and published *in vitro* ^18^ data. In addition, and somewhat surprisingly, we found that unbiased simulations of Aβ:Abcg4 dimer interactions indicated that Aβ binds to the same cleft as desmosterol, despite having greater than ten times the molar mass. While it is possible that modeling Abcg4 using ABCG5/ABCG8 incorporates a mechanistic bias into our model (for example, sterol binding), it is important to note that selection of that template was unbiased, as was the preference for desmosterol into the binding site. In addition, no loops or gaps in the sequence were needed to achieve a very high quality model (92^nd^ percentile, N = 27675, 0 Å–99 Å based on MolProbity score). Finally, while this paper was in revision, the structure of Abcg2 homodimer was published^57^. We also used this model as template, but our findings remain robust and unchanged (data not shown). This Aβ/desmosterol interaction was confirmed *in vivo* since desmosterol inhibited the Abcg4-mediated transport of [^3^H]Aβ_1-40_ in WT, but not in *Abcg4*-KO mice. The interaction between Aβ and desmosterol with Abcg4 suggests the complex may be a target for drug discovery via identification of Abcg4 modulators using computational tools and homology modeling as we have reported previously^58^.

Interestingly, the Km for the Aβ/Abcg4 interaction indicated a tighter affinity of Aβ for Abcg4 (350 pM) than reported for Abcb1 (12.5 µM)^59^. This could be explained by the prediction of two Aβ binding sites on the Abcg4 dimer, obtained by docking simulations. There may be functional cooperation between several ABC transporters ensuring a proper restriction of Aβ brain entry at the BBB level; we obtained similar Cl_up_ values in WT and *Abcg4*-KO mice as well as in WT and Abcb1/Abcg2-KO mice^18^. In *Abcb1/Abcg2*-KO mice, the efflux of Aβ may be offset by Abcg4 and in *Abcg4*-KO mice, the efflux of Aβ may be offset by P-gp and Abcg2. This concept of functional cooperation between different ABC transporters to efflux a substrate has been shown for sorafenib, a dual substrate of Abcb1 and Abcg2. Indeed, the brain clearance of sorafenib, measured using the brain efflux index (BEI) method, was the same in WT and *Mdr1a/b* ^−/−^ mice (without alteration of Abcg2 expression in *Mdr1a/b* ^−/−^ mice), but was 2- and 2.5-fold decreased in *Abcg2*- and *Abcb1/Abcg2*-deficient mice, respectively^60^. Similarly, in agreement with our desmosterol data, it has been shown that mouse brain desmosterol levels were unchanged in *Abcg4*-KO mice (without modification of the *Abcg1* mRNA level), whereas it was increased 1.4-fold and 2.4-fold in *Abcg1-* and *Abcg1/Abcg4* double KO mice, respectively^17^.

A link between AD and ABCG4 has been described, all of which involve non-BBB sites^21,22,26^. Hence, mice lacking Abcg4 have been found to have a defect in associative fear memory and an over-expression of ABCG4 has been observed in the brain of AD patients^25,61^. Our group was the first to point out a link between BBB, ABCG4 and AD by evidencing an over-expression of Abcg4 in young 3xTg-AD mice brain microvessels (3 months)^51^. Our present data, by demonstrating that Abcg4 can efflux Aβ through the mouse BBB, provides an explanation for the observation that 3-months’ aged 3xTg-AD mice could efflux more Aβ than WT, off-setting the increased RAGE-mediated influx of Aβ observed at the same time in 3xTg-AD mice^51^, in the face on an unchanged Cl_up_ of [^3^H]Aβ_1–40_ compared with WT mice^51^.

There is an indirect link between ABCG4 and AD, based upon the sterol efflux function of ABCG4^17^ and the sterol-AD relationship^22,62–65^. In particular, several studies have highlighted the strong interrelations existing between cholesterol and Aβ levels, although the mechanisms of this relationship are still not well understood^62,66,67^. Brain desmosterol was increased in a mouse model of AD (APPSLxPS1mut) compared with WT^68^. On the contrary, a decrease of the desmosterol level in brain tissues of AD patients compared to those of age-matched control patients has also been observed^69^. This decrease seems to be independent of the conversion rate of desmosterol to cholesterol^70^. Our results suggest a role of ABCG4 in the luminal BBB efflux of desmosterol, agreeing with previous data showing a role for Abcg4 in the efflux of desmosterol from other brain cells^17^. An increase of the ABCG4 expression and/or function in AD, as observed in AD patients and in 3xTg-AD mice^25,51^, could decrease the brain levels of desmosterol. Further studies are required to explore these mechanistic pathways.

These data are of a great importance, since they reveal 1) that another ABC protein (besides ABCB1 and ABCG2^71^) is involved in the clearance of Aβ at the luminal side of mouse BCECs and 2) that desmosterol can also be pumped out of the brain by an ABC protein located at the luminal side of the BCECs.

In conclusion, we provide evidence, for the first time, that Abcg4 functions at the luminal surface of mouse BCECs, where it can efficiently efflux Aβ and desmosterol. In addition, desmosterol may be a competitive inhibitor of Aβ efflux (or vice versa) and this may be part of the mechanistic link between disordered sterol metabolism and AD. Our data reveal ABCG4 as a new actor in the transport of Aβ at the BBB level and therapies to increase its function may mitigate the decreased clearance rate of Aβ seen in late-onset AD.

## Materials and Methods

### Animals

*Abcg4*-KO mice were produced in the Patel laboratory at the Medical University of South Carolina as described in *the Abcg4-KO mice line establishment* section (see below). *Abcg4*-KO mice were maintained and used in the animal house of the Faculty of Pharmacy of Paris (Paris Descartes University). *In vivo* experiments were performed with 8–12 weeks old mice. Wild-type C57BL/6 and *Abcg4*-KO mice had free access to standard laboratory food and water and were kept on a 12-hour light-dark cycle at 22 ± 3 °C. All studies involving animals and their care were performed according to the National Research Council’s guide and complied with the Ethical rules of the European directive (210/63/EU) for experimentation with laboratory animals. All experimental protocols were approved by the ethics review committee of Paris Descartes University (approval n° 12–181), the Animal Care and Use Committees of the Medical University of South Carolina, Charleston, SC, and the Medical College of Wisconsin, the IACUC for the Clement J. Zablocki VAMC, Milwaukee, WI and the IACUC at the University of Cincinnati, OH.

### Reagents

[^3^H]Aβ_1–40_ (20 Ci/mmol) and [^3^H]desmosterol (10 Ci/mmol) were purchased from Hartmann Analytic (Braunschweig, Germany), [^14^C]sucrose (435 mCi/mmol), liquid scintillation cocktails Ultima Gold XR® and Solvable® were purchased from PerkinElmer Life Sciences (Courtaboeuf, France). L-thyroxine (T_4_), probucol, desmosterol, Triton X-100, human serum, and bicinchoninic acid (BCA) assay kits were obtained from Sigma-Aldrich (Saint-Quentin-Fallavier, France). All the other chemicals were commercial products of reagent grade.

### Brain micro-vessel isolation, mRNA extraction and reverse transcription

Mouse brain was collected and the olfactory bulbs, cerebellum, thalamus, white matter, hippocampus, striatum, meninges and large superficial blood vessels were removed. For each sample, six cortices were pooled and immersed in Hank’s Balanced Salt Solution containing 1% Hepes (HBSS + 1% Hepes buffer) and centrifuged at 600 g for 5 min at 4 °C. After discarding the supernatant, the pellet was homogenized in HBSS + 1% Hepes buffer containing Liberase DL (37.5 µg/mL) and DNase I (20 U/mL) and incubated for 45 min at 37 °C. The resulting homogenate was centrifuged at 600 g for 5 min at 4 °C and the supernatant discarded. The pellet was homogenized in HBSS + 1% Hepes buffer containing 18% bovine serum albumin (BSA) and centrifuged at 2000 g for 15 min at 4 °C. After discarding the supernatant, the pellet was homogenized in HBSS + 1% Hepes buffer containing 1% BSA. The homogenate was passed through a 100 µm mesh cell strainer then through a 10 µm mesh nylon filter. The fraction retained on the 10 µm mesh nylon filter was collected, suspended in HBSS + 1% Hepes buffer containing 1% BSA and centrifuged at 600 g for 5 min at 4 °C. The pellet containing the brain microvessels was washed in HBSS + 1% Hepes buffer and centrifuged again at 600 g for 5 min at 4 °C. The supernatant was discarded; the pellets were re-suspended in RLT buffer containing β-mercaptoethanol and stored at -80 °C.

Total RNA was extracted from the micro-vessel samples using RNeasy Plus Micro Kit (Qiagen) according to the manufacturer’s instructions and treated with DNase I to remove genomic DNA. For each sample, 350 ng of extracted RNA were converted to cDNA using SuperScript® II Reverse Transcriptase (Invitrogen, ThermoFisher Scientific, Waltham, MA, USA) according to the manufacturer’s instructions and stored at −80 °C.

### Abcg4-knockout mice line establishment

#### Targeting vector construction and generation of knockout mice

A mouse genomic bacterial artificial chromosome (BAC) library (CitbCJ7, ES cell line/129 Sv, Research Genetics, Inc., Huntsville, AL, USA) was screened by using primers designed from the sequences of mouse *Abcg4* cDNA and a positive BAC clone was used as a template to amplify genomic DNA fragments of *Abcg4*. Long-fragment polymerase chain reaction (PCR) was performed using Expanded Long Template PCR system kit (Roche Applied Science, Indianapolis, IA, USA). An approximately 1.7 kb ‘long-arm’ genomic fragment containing exon 6 to partial exon 7 was inserted in-frame into the coding sequence for enhanced green fluorescent protein (pCRII, Invitrogen) and the exon 6–exon 7-eGFP fusion fragment (5’ arm, ‘short-arm’) was cloned into cloning site A of OSDUPDEL vector (supplied by Dr. Nobuyo Maeda, University of North Carolina) and verified by sequencing. The ‘long-arm’ genomic fragment, containing partial exon 9 to exon 14 (~4.2 kb), was cloned into cloning site B; homologous targeting would result in deletion of remaining exon 7, intron 7, exon 8 and intron 8 (Fig. 8A). The ES cell targeting and micro-injection of positive clones was performed by the transgenic core at the Medical University of South Carolina. ES cells (129/SvEvTac-cell line) were electroporated with the linearized targeting vector DNA and cultured on sub-confluent embryonic fibroblasts. Transfected cell colonies were selected by culturing cells in medium containing 200 µg/mL G418 (Life Technologies, Rockville, MD, USA). Out of 79 colonies screened by PCR, 18 clones were identified containing a homologously targeted 5’ arm, of which 11 clones were confirmed by Southern blotting. Positively targeted ES cells were microinjected into C57BL/6 J blastocysts and transplanted into pseudopregnant recipients. Three chimeric male mice (agouti coat color) were isolated and bred with C57BL/6 J mice, only one of which led to germ-line transmission. Heterozygous offspring mice were back-crossed to C57BL/6 J mice (N > 16) to produce disrupted line and inter-crossed to generate knockout mice (Fig. 8B).

#### Genotype: PCR analysis

Genotyping of mice was performed using PCR on mouse tails DNA (data not shown) using four primers. The Mo-ABCG4-WT-f (5′-CTGCCCTCCCTTATCAATC-3′) and Mo-ABCG4-WT-r (5′-TATCACAAGCCAGCCTTCTCGG-3′) primers were used to detect a 423 bp fragment from the WT allele. The Mo-ABCG4-Mut-f (5′-CTGCCCTCCCTTATCAATC-3′) and Mo-ABCG4-Mut-r (5′- TTGCTCACCATGGTGGCGACCGGTGG-3′) primers were used to detect a 400 bp fragment from the mutant allele. The PCR was performed using GoTaq® Green Master Mix (Promega, Madison, WI, USA). The program consists of a denaturation step at 95 °C for 10 min, followed by 40 cycles of denaturation (95 °C for 30 s), annealing (60 °C for 30 s) and extension (72 °C for 45 s) steps. The final extension at 72 °C for 7 min ends the program.

#### Southern blot analysis

DNAs were digested with *Bgl I*, subjected to electrophoresis in a 0.8% agarose gel and transferred to nylon membrane. Southern blot filters were hybridized with a ~800 bp PCR fragment from intron 2 to intron 5 (containing exons 3, 4 and 5), using a non-radioactive detection system (Dig labeling kit, Roche) (Fig. 8B).

#### Northern blot analysis

Isolation of total RNA and Northern blot analyses were performed as previously described^72^, the probe contained cDNA spanning exon 4–9 (Fig. 8C).

#### Sequencing of Abcg4 mRNA

Total RNA was extracted from mouse brain of WT and *Abcg4*-KO mice using RNeasy Lipid Tissue Mini Kit (Qiagen) according to the manufacturer’s instructions. Total RNA (500 ng) was reverse transcribed into cDNA in a final volume of 20 μL. The mixture consisted of 500 ng total RNA, 500 μM of each dNTP, 10 mM DTT, 1.5 μM random hexanucleotide primers, 20 U RNAse in ribonuclease inhibitor, and 100 U SuperScript II reverse transcriptase (Invitrogen). Hexamers were annealed at 25 °C for 10 min, products were extended at 42 °C for 30 min, and the reaction was terminated by heating to 99 C for 5 min and quick-chilling to 4 °C. The cDNA was then amplified by PCR using GoTaq® Green Master Mix (Promega). The PCR products were purified using NucleoFast® 96 PCR Clean Up kit (Macherey-Nagel, Hoerdt, France) according to the manufacturer’s instructions. The *Abcg4* mRNA was sequenced in WT and *Abcg4*-KO mice by Sanger sequencing using the Applied Biosystems 3730xl DNA Analyzer (ThermoFisher Scientific).

Northern analyses (Fig. 8C) showed that much shorter transcripts were detected compared to normal Abcg4. To characterize the transcripts, we performed RT-PCR of RNA extracted from brains of KO and WT mice. However, when we performed RT-PCR using primers forward primers in exons 4, 5 or 6 with the reverse primer in exon 10, we detected much shorter products in the *Abcg4*-KO brain compared to WT (Fig. 8D). Sequencing of these products revealed that in the knockout brains, there was a consistent skipping of exons 7–9, with exon 6 spliced to exon 10. A minor species was also detected (arrow, Fig. 8C) which showed exon 5 was spliced to exon 10. In silico translation of these products leads to frame-shift and premature chain termination after exon 6 for mRNA where exon 6 is spliced to exon 10 (major products). However, for the smaller exon 5-exon 10 spliced products, there is no frame-shift and a theoretical Abcg4 protein missing exons 6–9 sequences (missing 177 amino acids) can result in a ~53 kd protein, but is missing 36 of 50 peptides that are part of the antigen used to make the Abcg4 antibody as well as the critical Walker B and the signature C motifs (see Supplement). While this mutant protein is not expected to function (due to the loss of the critical motifs), a deleterious effect from a ‘toxic’ protein seems unlikely as the *Abcg4*-KO mice are very normal, fully fertile and exhibit no neurological defects even when >8 months old. Additionally, our blood brain barrier permeability studies (See Supplement) showed no differences between WT and KO mice.

Primer pairs for amplification and sequencing of *Abcg4* exons were designed based on *Abcg4* reference sequence (Genbank: NM-138955.3, Table 1).

**Table 1:** Sequence of primers used for *Abcg4* mRNA RT-PCR and sequencing.

Forward primer (5′-3′)	Reverse primer (5′-3′)	Amplified exon
TGAGGACCTTCCGCAAGATG	GCTGGTGGCAAATGTGTGGCTT	4 to 10
CCTGAAGCTGAGTGAGAAGCA	GCTGGTGGCAAATGTGTGGCTT	5 to 10
TGCTCTGGAACTGGTCAAC	GCTGGTGGCAAATGTGTGGCTT	6 to 10

### Quantitative RT-PCR

The mRNA levels of ABC transporter genes (*Abca1*, *Abcb1*, *Abcg1*, *Abcg2* and *Abcg4*) were determined in WT and *Abcg4-*KO mice by quantitative real-time PCR (qRT-PCR) using Power SYBR® Green PCR Master Mix (ThermoFisher Scientific) on an ABI Prism 7900 HT sequence detection system (Applied Biosystems, Foster City, CA, USA). All the primers used [Table 2] were tested upstream on positive controls using SYBR Green fluorescence detection. The *Abcg4* primers were designed in exons 2 and 3 which belong to the non-mutant sequence of the *Abcg4* gene. The expression levels of all the transcripts were normalized to the expression levels of the housekeeping gene β-actin in the same tissue. The comparative Ct method with the formula 2^−ΔΔCt^ (where ΔΔCt = ΔCt_KO_ − ΔCt_WT_) was used to calculate the relative expression of each gene.

**Table 2:** Sequence of primers used for qRT-PCR.

Gene	Forward primer (5′-3′)	Reverse primer (5′-3′)	Length (bp)
*Abca1*	AGCTGGGAAGTCAACAACTTTCA	GCCCATGTTCTGGTGTACTTCAT	120
*Abcb1*	AAGCGACTCCGATACATGGTTT	AGTGCTCCGGTGGTGTTTTTAGG	85
*Abcg1*	GCTGCCTCACCTCACTGTT	CTCTCGTCTGCCTTCATCCTTC	81
*Abcg2*	TGGCTGTCCTGGCTTCAGTAC	AGTGCTGTTGTCCGTTACATTGA	116
*Abcg4*	CGCTCTGCTGTGGACATCG	CTCGGCGACAGAACTTACCAG	119

### Confocal imaging of mouse cortex cryosections

Brains of WT mice were fresh-frozen in isopentane at −45 °C and staining was performed on 20 µm thick cryosections. Sections were fixed in methanol (10 min at −20 °C) and blocked in PBS, 10% Goat serum, 1% BSA, 0.1% Triton for 1 h at RT. They were incubated overnight at 4 °C in a mixture of mouse anti-Pgp (C219 clone, Enzo Life Sciences, Villeurbanne, France) and rabbit anti-ABCG4 (sc-33825, Santa Cruz Biotechnology, Heidelberg, Germany) antibodies diluted 1/50 and 1/100 in PBS, respectively. Sections were then reacted with Goat anti-rabbit Alexafluor 488 and Goat anti-Mouse IgG1 Alexafluor 555 (Thermo Fisher Scientific) diluted 1/300 for 2 h at RT. Nuclei counterstaining was performed by incubation in Topro3 (ThermoFisher Scientific) diluted 1/500 for 15 min at RT. Thorough PBS washing was performed after each incubation step. Sections were mounted in 90% glycerol in PBS and viewed in a Leica TCS SP2 confocal microscope (Leica microsystems SAS, Nanterre, France) equipped with a ×40 objective with NA = 1.0. Images were taken with the same acquisition parameters for all conditions and no detectable signal was observed when primary antibodies were omitted.

### In situ brain perfusion technique

#### Surgical procedures and Perfusion

Mice were anesthetized by intra-peritoneal injection of a mixture of ketamine and xylazine at 140:8 mg/kg. The right common carotid artery was ligated on the heart side and the right external carotid artery was ligated at the level of the bifurcation. A catheter was inserted into the right common carotid artery. The perfusion liquid was contained in a syringe placed in an infusion pump and connected to the catheter. Before the perfusion, the thorax of the animal was opened, the heart was removed and perfusion started immediately at flow of 2.5 mL/min. The perfusion fluid was Krebs bicarbonate-buffered physiological saline (128 mM NaCl, 24 mM NaHCO_3_, 4.2 mM KCl, 2.4 NaH_2_PO_4_, 1.5 mM CaCl_2_, 0.9 mM MgCl_2_ and 9 mM D-glucose).The solution was gassed with 95% O_2_−5% CO_2_ to bring the pH to 7.40 and warmed to 37 °C in water bath. Each mouse was perfused for 60 s with [^3^H]Aβ_1–40_ (0.2 µCi/mL) or [^3^H]desmosterol (0.2 µCi/mL). [^14^C]sucrose was used as a vascular marker of the physical integrity of the BBB and co-perfused with the tritiated substrate. The brain uptake of tritiated tracers was also measured in the presence of unlabeled substrates [probucol 10 µM, desmosterol 20 µM orT_4_ 10 µM], dissolved in their respective vehicles [DMSO ( < 0.5% in final perfusion fluid) or H_2_O/NaOH]. The experiment was terminated by decapitating the mouse. The right cerebral hemisphere and aliquots of the perfusion fluid were sampled in tared vials, weighted, digested in 2 mL of Solvable® solubilizer (PerkinElmer, Courtaboeuf, France) at 50 °C and mixed with 9 mL of Ultima Gold XR® scintillation cocktail (PerkinElmer). Labeled compounds were counted on a Tri-Carb® (PerkinElmer) counter with the appropriate dual (^14^C/^3^H) quenching curves and standards to measure disintegration per minute (dpm) in samples.

#### Calculation of the BBB transport parameters

All the calculations were done as described previously^41^. The brain vascular volume of each animal (V_vasc_, µL/g) was estimated using the distribution volume of [^14^C]sucrose, which does not measurably cross the BBB during short perfusions, by dividing the amount of [^14^C]sucrose (dpm/g) in the right brain hemisphere by the concentration of [^14^C]sucrose in the perfusion fluid (dpm/µL). All the values were under 20 µL/g, indicating that the BBB was not altered during these experiments^40^. The brain uptake clearance (Cl_up_, µL/g) is the ratio between the apparent tissue distribution volume (V_brain_, µL/g) of [^3^H]Aβ_1–40_ or [^3^H]desmosterol and the perfusion time (s). The V_brain_ was calculated from the amount of radioactivity in the right brain hemisphere using the following equation:

$${{\rm{V}}}_{{\rm{b}}{\rm{r}}{\rm{a}}{\rm{i}}{\rm{n}}}={{\rm{X}}}_{{\rm{b}}{\rm{r}}{\rm{a}}{\rm{i}}{\rm{n}}}/{{\rm{C}}}_{{\rm{p}}{\rm{e}}{\rm{r}}{\rm{f}}}$$

where X_brain_ (dpm/g) is the calculated amount of tritiated tracer in the right cerebral parenchyma and C_perf_ (dpm/µL) is the concentration of tritiated tracer in the perfusion fluid. X_brain_ is obtained by this equation:

$${{\rm{X}}}_{{\rm{b}}{\rm{r}}{\rm{a}}{\rm{i}}{\rm{n}}}{\rm{=}}{{\rm{X}}}_{{\rm{t}}{\rm{o}}{\rm{t}}}{\rm{-}}({{\rm{V}}}_{{\rm{v}}{\rm{a}}{\rm{s}}c}{\rm{\times }}\,{{\rm{C}}}_{{\rm{p}}er{\rm{f}}})$$

where X_tot_ (dpm/g) is the total amount of tritiated tracer measured in the brain tissue sample (vascular and extravascular).

### Abcg4 dimer structural modeling

The mouse sequence of Abcg4 (UniProtKB Q8K4E1) was submitted to the MobBase modeling server^73^ to create a homology model of an Abcg4 monomer. ModBase selected the structure of ABCG5/ABCG8 (PDB code 5do7A) as the template and produced 5 Abcg4 models including one that spanned target sequence from 82–641 (corresponding to template sequence 66–645). This model was selected and optimized by adding missing hydrogen atoms, tuning side-chains, and energy minimizing using default settings in Yasara Structure (www.YASARA.org). Model quality and optimization were assessed using MolProbity^45,46^. The final Abcg4 homodimer was created by aligning an Abcg4 monomer to both ABCG5 and ABCG8 using the align instruction in PyMOL (www.pymol.org). The resulting Abcg4 homodimer was used for docking studies and is available at www.somelink.edu.

### Docking

#### Sterols

The optimized Abcg4 homodimer was submitted to the SwissDock docking server^47^ as the binding target after removing the intracellular nucleotide binding domains in order to meet server-imposed size constraints. Both desmosterol and cholesterol were allowed to dock freely across the entire dimer as an unbiased approach to identify potential binding sites.

#### Aß

After removing the nucleotide binding domains, the Abcg4 homodimer was submitted to the GRAMM-X Protein-Protein Docking Web Server^48,49^ as the receptor and Aβ as the ligand (PDB code 1aap, chain A) and the top ten predictions were returned.

### Statistical analysis

Results are expressed as means ± S.D. Groups were compared by Student’s unpaired *t* test or by two-way analysis of variance (ANOVA) with interaction (Treatment (Inhibitor/no inhibitor) and genetics (WT/*Abcg4*-KO mice)) followed by a Bonferroni post-test, when appropriate. Differences were considered to be statistically significant when *p* < 0.05.

### Materials and correspondence

Correspondence and material requests should be addressed to Fanchon Bourasset and Shailendra B. Patel.

## Electronic supplementary material

Supplementary informations
