# Supplementary material for: Expression and function of Abcg4 in the mouse blood-brain barrier: role in restricting the brain entry of amyloid-β peptide
Source: Sci Rep. 2017 Oct 17;7:13393. doi: 10.1038/s41598-017-13750-0 (PMC5645361; doi:10.1038/s41598-017-13750-0)
Supplement: Supplementary file 1 — Supplementary informations [file 41598_2017_13750_MOESM1_ESM.pdf]

Expression and function of Abcg4 in the mouse blood-brain barrier: role in restricting the brain entry of amyloid-β peptide

Agnès Dodacki[1,2,3], Matthew Wortman[4], Bruno Saubaméa[1,2,3], Stéphanie Chasseigneaux[1,2,3], Sophie Nicolic[1,2,3], Nathalie Prince[1,2,3], Murielle Lochus[1,2,3], Anne-Laure Raveu[1,2,3], Xavier Declèves[1,2,3], Jean-Michel Scherrmann[1,2,3], Shailendra B. Patel[4,*] and Fanchon Bourasset[1,2,3,*]

[1]Variabilité de Réponse aux Psychotropes, INSERM, U1144, Paris, France
[2]Faculté de Pharmacie de Paris, Université Paris Descartes, UMR-S 1144, Paris, France
[3]Université Paris Diderot, UMR-S 1144, Paris, France
[4]Division of Endocrinology, Diabetes and Metabolism, University of Cincinnati, Cincinnati, OH, USA

Corresponding authors:
*To whom correspondence should be addressed:
Fanchon Bourasset, PharmD, PhD., Faculté de Pharmacie, Université Paris Descartes, 4 avenue de l'Observatoire, 75006 Paris, France.
Tel: +33 1 53 73 98 59
E-mail: fanchon.bourasset@parisdescartes.fr
or,
Shailendra Patel, MD, PhD. Division of Endocrinology, Diabetes and Metabolism, 231 Albert Sabin Way, Cincinnati, OH 45219-0547, USA
Tel : +1 513 558 4444
E-mail : sbpatel@ucmail.uc.edu

Supplementary figures

The following are the images used to create the composite figure 8.

1. Southern blot

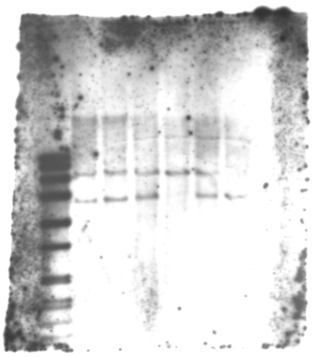

The lanes are as shown in Figure 8.

2. Northern blot-

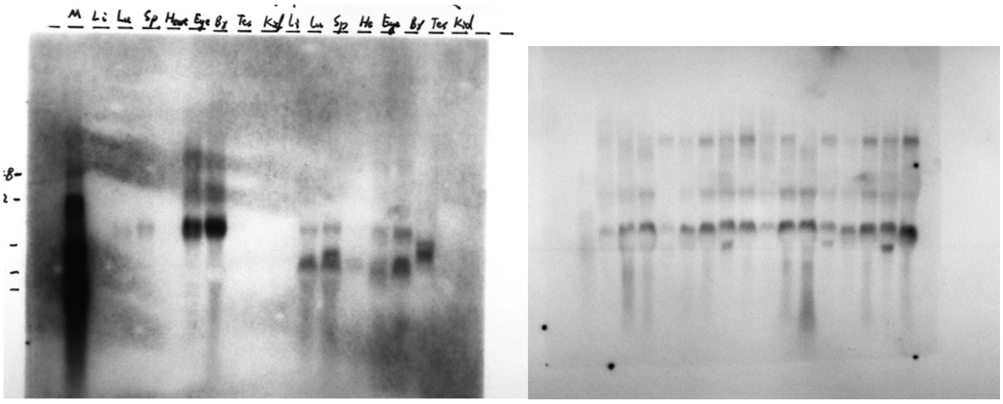

The left-hand panel was probed with the Abcg4 probe, the filter stripped and re-probed with a probe for actin. (right hand panel).

3. <u>RT-PCR for exon walking</u>

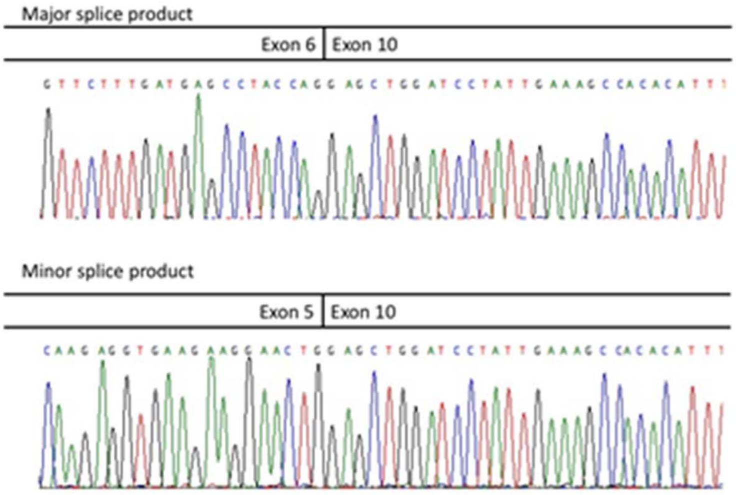

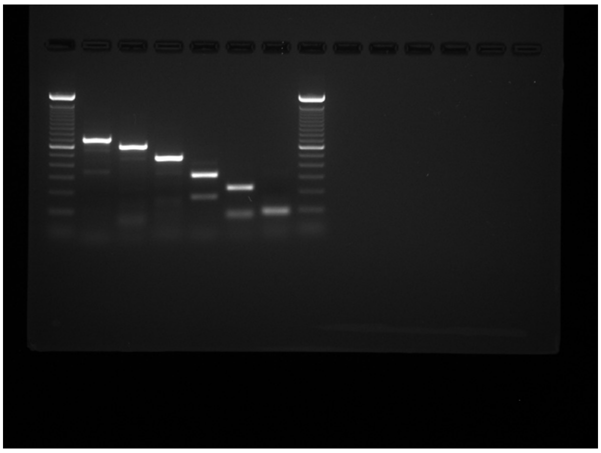

4. <u>Sequence analyses of major and minor splice products</u>

The major splice products from RT-PCR of Abcg4 KO mRNA (Fig 8, lanes 4, 5 and 6) from brains were sequenced and a representative trace is shown (top panel)- all showed splicing of Exon 6 to Exon 10 (that leads to frame-shift and premature chain termination). The minor smaller PCR product (Fig 8, lane 4, arrow) showed splicing of Exon 5 to Exon 10.
Although Exon 5 spliced to Exon 10 can lead to an in-frame splice, translation of this mRNA shows a deletion of 177 aa, which include the Walker B and Signature C motifs necessary for function. Clustal W alignment of Abcg4 WT polypeptide (top panel) to the Exon 5 to Exon 10 spliced mRNA translated polypeptide (bottom panel), performed by MacVector is shown below.

The neutral amino acids are grey, the basic residues blue, the acidic residues red. The deletion of 177 AA was seen between residues 180 and 357

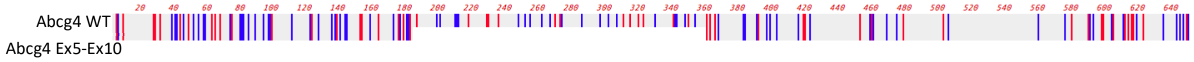

5. <u>Blood Brain Barrier permeability of WT and Abcg4 KO mice</u> (measure of the brain volume of distribution of sucrose)

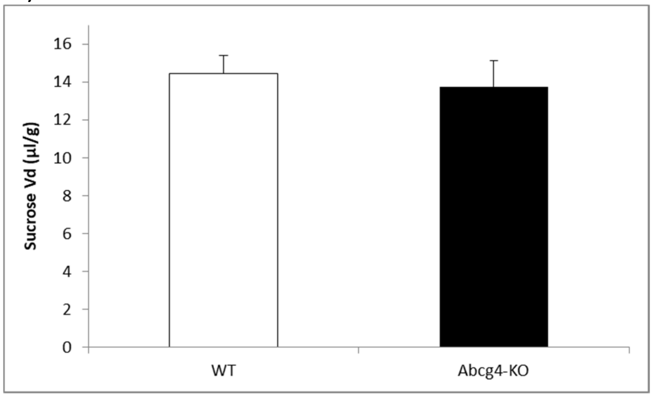

N = 12,
t-test: NS (p = 0.39)
